# High mesothelin expression in advanced lung adenocarcinoma is associated with *KRAS* mutations and a poor prognosis

**DOI:** 10.18632/oncotarget.3429

**Published:** 2015-04-13

**Authors:** Anish Thomas, Yuanbin Chen, Seth M. Steinberg, Ji Luo, Svetlana Pack, Mark Raffeld, Zied Abdullaev, Christine Alewine, Arun Rajan, Giuseppe Giaccone, Ira Pastan, Markku Miettinen, Raffit Hassan

**Affiliations:** ^1^ Thoracic and GI Oncology Branch, National Cancer Institute, National Institutes of Health, Bethesda, MD, USA; ^2^ Biostatistics and Data Management Section, Center for Cancer Research, National Institutes of Health, Bethesda, MD, USA; ^3^ Laboratory of Cancer Biology and Genetics, National Cancer Institute, National Institutes of Health, Bethesda, MD, USA; ^4^ Laboratory of Pathology, Center for Cancer Research, National Cancer Institute, National Institutes of Health, Bethesda, MD, USA; ^5^ Laboratory of Molecular Biology, Center for Cancer Research, National Cancer Institute, National Institutes of Health, Bethesda, MD, USA; ^6^ Lombardi Cancer Center, Georgetown University, Washington DC, USA

**Keywords:** mesothelin, non-small cell lung cancer, KRAS, EGFR

## Abstract

Mesothelin is a cell surface glycoprotein which is highly expressed in several epithelial cancers and may have a role in cell adhesion and metastases. In this study, we used prospectively obtained clinical and pathological data to characterize mesothelin expression in advanced lung adenocarcinoma. Tissue was obtained from patients who underwent molecular profiling of potentially actionable genes on a trial of molecular profiling and targeted therapies in advanced thoracic malignancies. We immunohistochemically evaluated the intensity, and the percentage of cells expressing mesothelin in 93 advanced lung adenocarcinomas. The evaluation was blinded for molecular data and outcome. Mutations of *EGFR, KRAS, BRAF, AKT1, PIK3CA* and *HER2* were assessed by pyrosequencing; *HER2* amplification and *ALK* translocation were assessed by fluorescence in situ hybridization. 53% of advanced lung adenocarcinomas expressed mesothelin to some degree; high mesothelin expression, defined as mesothelin positivity in more than 25% of cells, was found in 24% of patients. High mesothelin expression was associated with inferior survival (median 18.2 months vs. 32.9 months; *P* = 0.014). High mesothelin expression was strongly associated with mutant *KRAS* (*P* < 0.0001) and wild-type *EGFR* (*P* = 0.002). Our results provide strong rationale to explore anti-mesothelin targeted therapies in advanced lung adenocarcinoma especially in the *KRAS*-mutant subgroup.

## INTRODUCTION

Lung cancer is the leading cause of cancer-related death worldwide, accounting for more than one million deaths every year. [1, 2] Non-small-cell lung cancer (NSCLC) constitutes approximately 85% of lung cancers and about 40% of patients with newly diagnosed NSCLC have advanced disease. [1] In the past decade, the standard of care for patients with advanced disease was platinum-based chemotherapy, which improved survival, quality of life and symptom control compared with supportive care. [3] However, the median overall survival is only about a year; only 3.5% of patients are alive five years after diagnosis. Chemotherapy is also associated with high morbidity. [4] In recent years, identification of “druggable” oncogenic alterations such as mutations in *EGFR* and *ALK* translocations, and development of drugs that specifically target these mutations have led to a substantial improvement in the prognosis of patients with advanced lung cancer. [5] However, “druggable” alterations have been detected in less than half of all advanced NSCLC patients. [6] Mutations in the *KRAS* oncogene, for example, accounts for 20–30% of lung adenocarcinomas, yet no targeted agents are currently available. Hence there is an unmet need to develop new, effective and minimally toxic targeted therapies in advanced NSCLC.

Mesothelin is a 40-kDa cell surface glycoprotein that is present on normal mesothelial cells lining the pleura, peritoneum and pericardium. [7] Mesothelin expression in normal human tissues is observed only in a single layer of mesothelial cells lining the pleura, peritoneum and pericardium, surface epithelial cells of the ovary, tunica vaginalis, rete testis and the tonsilar and fallopian tube epithelial cells. [8] However, mesothelin is highly expressed in several cancers, including epitheloid mesotheliomas, pancreatic, biliary adenocarcinomas, gastric and ovarian cancers. [8–11] The high expression of mesothelin in cancers have prompted its therapeutic targeting using a variety of strategies including immunotoxins, monoclonal antibodies, antibody drug conjugates, vaccines and adoptive T cell therapy. [12–14] We recently demonstrated major and durable tumor regressions in chemotherapy-refractory patients with advanced epitheliod mesothelioma using the anti-mesothelin immunotoxin SS1P. [15]

Conceptually, SS1P and other mesothelin-targeted therapies might also confer efficacy in other tumor types that over-express mesothelin. Identifying these cancers could thus expand the therapeutic utility of these therapies. Mesothelin expression has been demonstrated in approximately 30–70% of lung adenocarcinoma. [16–20] However, these studies were retrospective, analyzed a limited number of samples, did not provide clinical information and did not study the patterns of expression in detail.

Given the paucity of data and the heterogeneous and conflicting results of prior investigations, we sought to determine the expression patterns and prognostic value of mesothelin in advanced lung adenocarcinoma and the association of mesothelin expression with other molecular alterations and clinico-pathologic variables. We demonstrate here that 24% of advanced lung adenocarcinoma express high levels of mesothelin, and that high mesothelin expression is associated with *EGFR* wild-type and mutant *KRAS* and, independent of the mutation status, is associated with decreased overall survival. Our results suggest that mesothelin targeted therapies could be useful in patients with *KRAS* mutant lung cancer, a subtype for which no targeted therapies are currently available.

## RESULTS

From February 2011 to December 2012, 272 patients with NSCLC enrolled and underwent molecular profiling in the pilot trial of molecular profiling and targeted therapies in advanced thoracic malignancies at the Center for Cancer Research, National Cancer Institute. [21] Two hundred and eleven had adenocarcinoma histology with 179 having advanced disease (stages III or IV) at diagnosis. Ninety three patients had adenocarcinoma histology, stage III or IV at diagnosis and had adequate FFPE samples available for further studies.

### Patient characteristics

The clinicopathological characteristics are summarized in Table 1. The median age of all patients was 61 years and 53 (57%) patients were female. The patient population was predominantly Caucasian (74%) and 35% were never-smokers. Oncogenic alterations in *EGFR*, *KRAS* mutations and *ALK* translocations were found in 25%, 29% and 11% patients respectively.

**Table 1:** Demographic and clinico-pathologic characteristics (*n* = 93)

Variable	No. of patients (%)
Age, median (range) ≥ 60 <60	61 (24–82) 42 (45) 51 (55)
Sex Male Female	40 (43) 53 (57)
Race Asian Black Caucasian Hispanic	13 (14) 8 (9) 69 (74) 3 (3)
Smoking Never Ever	33 (35) 60 (65)
Stage at Biopsy III IV	7 (8) 86 (92)
EGFR mutation Negative Positive ex18G719A ex19del ex21L858R Not tested	70 (68) 20 (25) 1 (5) 8 (40) 11 (55) 3 (7)
KRAS mutation Negative Positive G12A G12C G12D G12V G13D G13Y Not tested	65 (70) 27 (29) 3 (11) 7 (26) 6 (22) 9 (33) 1 (4) 1 (4) 1 (1)
BRAF mutation Negative Positive Not tested	85 (91) 2 (2) 6 (6)
AKT1 mutation Negative Positive Not tested	54 (58) 0 39 (42)
PIK3CA mutation Negative Positive Not tested	52 (55) 2 (2) 39 (43)
HER2 mutation Negative Positive Not tested	35 (37) 0 58 (63)
HER2 amplification Negative Positive Not tested	83 (89) 2 (2) 8 (8)
*ALK* translocation Negative Positive Not tested	74 (80) 10 (11) 9 (9)

### Mesothelin expression in lung adenocarcinoma

The intensity and percentage of cells expressing mesothelin in advanced lung adenocarcinoma is shown in Table 2. Of the 93 tumors tested, any expression of mesothelin was observed in 49 (53%) and high expression in 22 (24%). Figure 2 shows representative images of mesothelin expression in lung cancers. Mesothelin expression was observed in the cytoplasm alone in 10 (20%), membrane alone in 14 (29%) and in both membrane and cytoplasm in 25 (51%).

**Figure 1:**
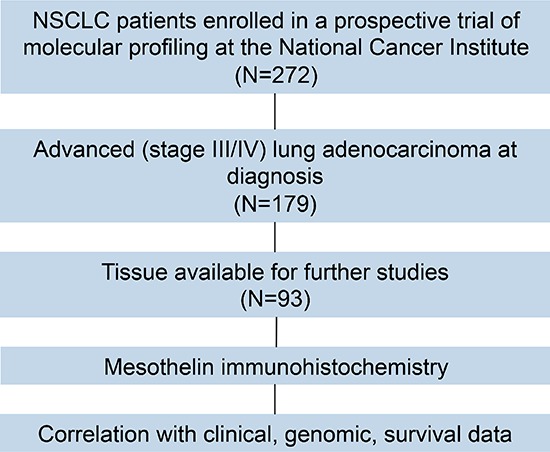
Flow diagram showing the study design

**Figure 2:**
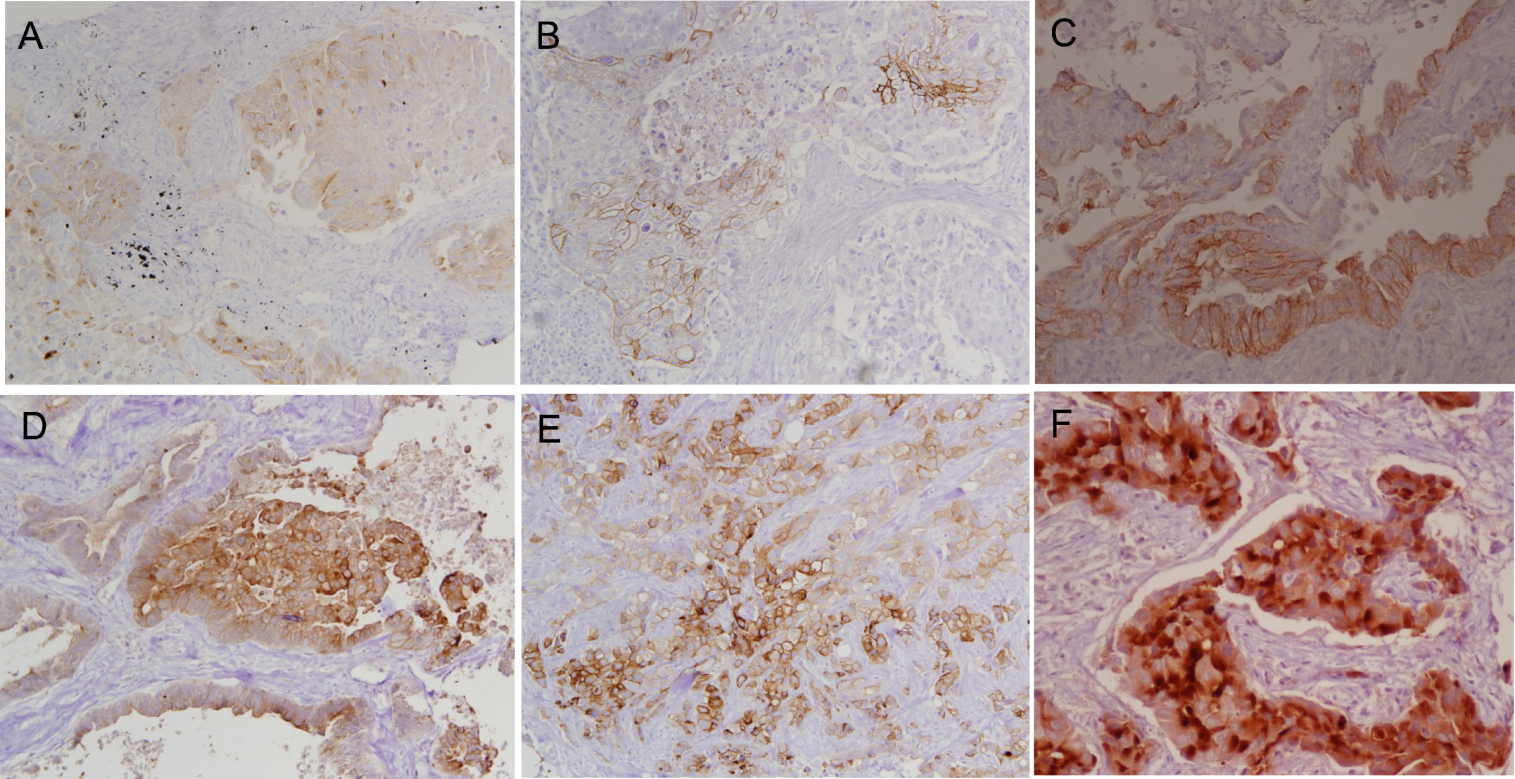
Tumor expression of mesothelin in lung adenocarcinoma was evaluated using immunohistochemistry Representative images are depicted (original magnification x400). Focal cytoplasmic immunostaining of 2+ intensity in 15% cells **A.**, membranous and cytoplasmic immunostaining of 2+ intensity in 1% cells **B.**, membranous immunostaining in of 3+ intensity in 30% cells **C.**, membranous and cytoplasmic immunostaining of 3+ intensity in 60% cells **D.**, membranous and cytoplasmic immunostaining of 3+ intensity in 80% cells **E.**, membranous and cytoplasmic immunostaining of 3+ intensity in 100% cells **F.**

**Table 2:** Mesothelin expression in advanced lung adenocarcinoma (*n* = 93)

Mesothelin expression	Percentage of mesothelin positive cells	1+	2+	3+	*n* (%)
Negative					44 (47)
Positive	≥ 1%	11	16	22	49 (53)
	>25%	4	4	14	22 (24)

### Association of mesothelin expression with clinico-pathological variables

**Figure 3:**
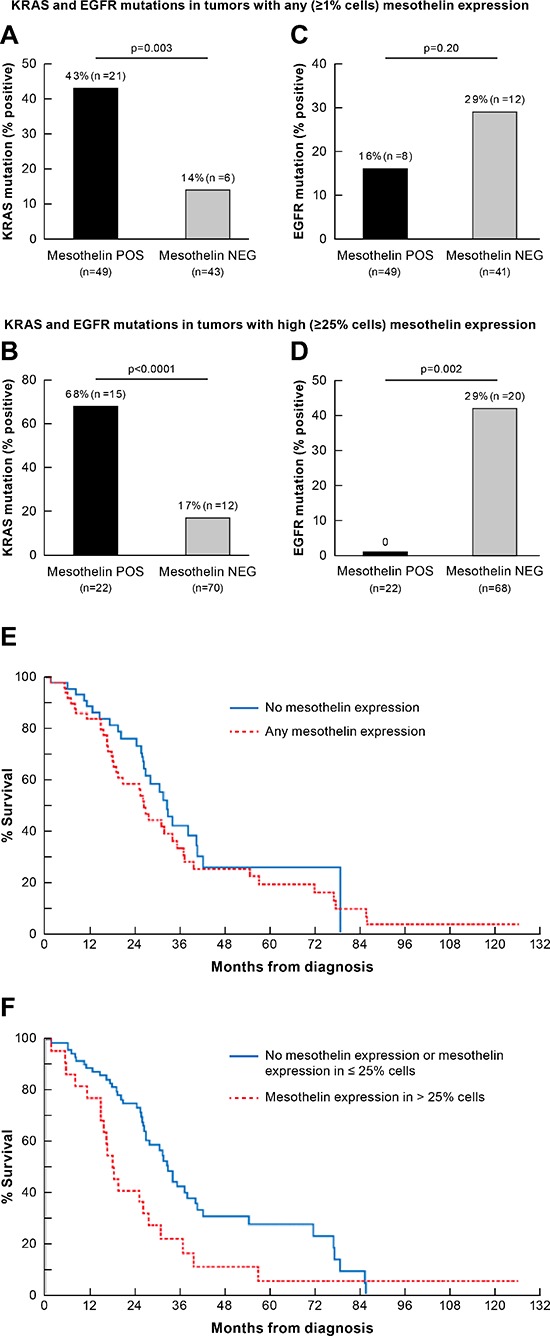
Association between mesothelin expression and *KRAS* and *EGFR* mutations and overall survival Association between *KRAS* mutation any mesothelin expression **A.** and high mesothelin expression (in more than 25% cells) **B.** Association between *EGFR* mutation any mesothelin expression **C.** and high mesothelin expression (in more than 25% cells) **D.** Overall survival of patients with any mesothelin expression compared with no mesothelin expression (median 32.4 months vs. 26.2 months; *p* = 0.29; **E.**). Overall survival of patients with high mesothelin expression (in more than 25% cells) compared with ≤ 25% or no mesothelin expression (median 18.2 months vs. 32.9 months; *p* = 0.014; **F.**).

The association between mesothelin expression and clinico-pathological characteristics in advanced lung adenocarcinoma is shown in Table 3. There was no association between mesothelin expression and age, sex, or race. There was a strong association between *KRAS* mutation and mesothelin expression. Twenty one of 49 (43%) of tumors that expressed at least some mesothelin (> = 1% cells) had a *KRAS* mutation whereas only 6 of 43 (14%) mesothelin negative tumors expressed this mutation (*P* = 0.003; Figure 3A). The association with *KRAS* mutation was stronger for high mesothelin expressors (> = 25% cells): 15 of 22 (68%) mesothelin positive tumors with high mesothelin expression had a RAS mutation compared with only 12 of 70 (17%) of mesothelin negative tumors (*P* < 0.0001; Figure 3B).

**Table 3:** Association between mesothelin expression and clinico-pathological characteristics of the advanced lung adenocarcinoma (*n* = 93)

	Any mesothelin expression (≥ 1% cells)			High mesothelin expression (in >25% cells)		
	Mesothelin positive (*n* = 49)	Mesothelin negative (*n* = 44)	*P* value	Mesothelin positive (*n* = 22)	Mesothelin negative (*n* = 71)	*P* value
Age, median (range)	61 (24–80)	61 (29–82)	0.67	61.5 (42–80)	61 (24–82)	0.62
Sex Male Female	19 30	21 23	0.41	10	30 41	0.81
Race Asian Black Caucasian Hispanic	7 4 37 1	6 4 32 2	0.97	0 2 20 0	13 6 49 3	0.08
Smoking Never Ever	17 32	16 28	1.00	3 19	30 41	0.02
Stage at Biopsy III IV	6 43	1 43	0.11	3 19	4 67	0.35
EGFR mutation Negative Positive	41 8	29 12	0.20	22 0	48 20	0.002
KRAS mutation Negative positive	28 21	37 6	0.003	7 15	58 12	<0.0001
HER2 amplification Negative positive	46 2	37 0	0.50	21 0	62 2	1.00
ALK translocation Negative positive	44 4	30 6	0.31	20 1	54 9	0.44

Mesothelin expression in more than 25% of cells was also significantly associated with smoking and wild-type *EGFR*. Tumors from 19 of 60 (32%) current or ex-smokers expressed mesothelin in more than 25% cells, whereas tumors from 3 of 33 (9%) never-smokers which expressed mesothelin (*P* = 0.02). Eight of 49 (16%) of mesothelin positive (> = 1% cells) patients had *EGFR* mutations compared to 12 of 41 mesothelin negative patients (*p* = 0.20; Figure 3C). None of the 22 mesothelin-expressing tumors with high mesothelin expression (>25% cells) had *EGFR* mutations compared to 20 with *EGFR* mutations among 68 mesothelin negative samples (*P* = 0.0024; Figure 3D). No association was found between mesothelin expression and the other molecular alterations evaluated: amplification of *HER2, ALK* translocation and mutations in *BRAF, AKT1, PIK3CA* or *HER2*. By logistic regression analysis, the association between *KRAS* mutation and high mesothelin expression was independent of co-variates including smoking, sex, stage at biopsy, and age.

### Mesothelin expression and survival

There was no difference in survival from diagnosis between patients with any mesothelin expression and patients with non-mesothelin expressing tumors. (median 32.4 months vs. 26.2 months; *P* = 0.29) (Figure 3E). However, patients with high mesothelin-expressing tumors had significantly shorter overall survival compared with patients with low or no mesothelin expression. (median 18.2 months vs. 32.9 months; *P* = 0.014) (Figure 3F). The median potential follow-up (from date of diagnosis until analysis, February 1, 2014) for all patients was 38.6 months. There was no association between the pattern of mesothelin expression (cytoplasmic vs. membranous) and survival.

A Cox proportional hazards regression model was used to evaluate the association between mesothelin expression and overall survival from date of diagnosis. The following covariates were included: age, smoking, *KRAS* and *EGFR* mutations. Mesothelin vs. non-mesothelin expression remained not significantly associated with survival after adjusting for clinical covariates. However, high mesothelin expression remained significantly associated with survival when adjusted for covariates. Specifically, in a model beginning with the parameters as stated above, backward selection resulted in a model including only high mesothelin expression (*P* = 0.015; Hazard Ratio (HR)=1.94; 95% confidence interval (CI), 1.14–3.30) and smoking history (*P* = 0.045; HR = 0.57; 95% CI, 0.33–0.99) as parameters retaining their joint statistical significance.

## DISCUSSION

In this study, we used prospectively obtained clinical and pathological data to characterize mesothelin expression in lung cancer. We evaluated the intensity, and the percentage of cells expressing mesothelin in tissue obtained from 93 patients with advanced lung adenocarcinoma who underwent molecular profiling for potentially actionable genes using a multi-platform approach. We found that approximately 50% of advanced lung adenocarcinomas express mesothelin and high mesothelin expression, defined as mesothelin positivity in more than 25% of cells, was associated with inferior survival. Importantly, we found that high-expression of mesothelin was strongly associated with mutant *KRAS* and wild-type *EGFR*.

In early-stage lung adenocarcinoma, a recent retrospective study found mesothelin expression in 69% of tumors. Patients with high mesothelin expression were more likely to have *KRAS* mutations, compared with patients with low expression [22]. In the advanced lung cancer setting, prior studies of mesothelin expression are retrospective, involved small number of patients and a very heterogeneous population. [16–20] In addition to being the largest series of prospective assessment of mesothelin expression in lung cancer, the strengths of our study include a uniform patient population, all of whom enrolled in a trial of tumor molecular profiling, availability of robust clinical, pathologic, immunohistochemical, and mutational data and long-term follow-up.

The mechanistic association of *KRAS* mutation to mesothelin is not delineated by the present study. The observed enrichment for mesothelin expression within the *KRAS* mutated lung adenocracinoma population could occur due to a direct or indirect regulatory relationship between the two proteins. Transcription enhancer factor (TEF-1) is known to be upregulated in tumors from a *KRAS* mutated, genetically engineered mouse model of lung cancer. [23] TEF-1 has also been shown to directly bind an upstream enhancer sequence in the mesothelin gene, causing upregulation of mesothelin transcription. [24] However, concordance of *KRAS* mutation and mesothelin expression have not been observed in mesothelioma and high grade serous ovarian cancers where robust mesothelin expression is nearly universal [8, 17] and *KRAS* mutation quite rare. [25, 26] By contrast, almost all pancreatic adenocarcinomas express both mesothelin and mutated *KRAS*. However, the temporal relationship of their expression in tumor development is not suggestive of a regulatory relationship since *KRAS* mutation is one of the earliest detectable changes in pre-malignant PanIN lesions while mesothelin expression does not occur until much later in adenocarcinoma development. [27, 28] These data from other tumor types suggest that *KRAS* mutation alone is neither necessary nor sufficient to universally induce mesothelin expression and that other pathways must also participate in regulation of mesothelin. Our data are consistent with these observations since complete concordance between *KRAS* mutation status and mesothelin expression was not observed in our study. Nevertheless, this does not preclude the possibility that there may be a direct regulatory relationship in some lung tumors. Further studies would be required to establish whether this does occur.

The results of this study and previous work by others provide strong rationale that anti-mesothelin targeted therapy should be explored as a therapeutic modality in advanced lung adenocarcinomas. Our prior studies have demonstrated that mesothelin mRNA and protein are present in a substantial number of lung adenocarcinoma cell lines and that SS1P, an anti-mesothelin recombinant immunotoxin, was cytotoxic to mesothelin expressing lung cancer cell lines with IC50 values ranging from 2 to 5 ng/mL. [29] We have recently shown that in patients with chemotherapy refractory malignant mesothelioma, SS1P in combination with pentostatin and cyclophosphamide leads to durable tumor regression. [15] These lines of compelling evidence suggest that SS1P may confer clinical activity in mesothelin-expressing lung adenocarcinomas. Several other mesothelin-targeted therapies including a vaccine [30], antibody drug conjugate [31] and a monoclonal antibody [32] are undergoing phase I/II clinical trials.

The current study provides a strong rationale to target mesothelin in advanced lung adenocarcinoma and suggest that clinical trials of mesothelin-directed therapies in lung cancer should focus on patients with *KRAS* mutations, the most commonly mutated oncogene in NSCLC and one which has proven intractable even in the era of targeted therapy.

## METHODS

### Patients

Patients were prospectively enrolled in a pilot trial of molecular profiling and targeted therapies in advanced thoracic malignancies (Figure 1) (ClinicalTrials.gov Identifier: NCT01306045). [21] Main eligibility criteria were histologically confirmed, advanced stage lung cancer and Eastern Cooperative Oncology Group Performance Status ≤ 2. Patients had to have biopsiable disease and be willing to undergo biopsy for molecular profiling or have formalin-fixed paraffin-embedded (FFPE) tissue blocks suitable for molecular profiling analysis. Patients underwent new tumor biopsies when possible but archival tumor samples were also used. Tumor samples were screened for mutations in *AKT1, BRAF, EGFR, HER2, KRAS, NRAS*, and *PIK3CA* by pyrosequencing; *HER2* amplification and *ALK* translocation were assessed by Fluorescence in situ hybridization (FISH). Patients who did not have a FFPE sample available for mesothelin immunohistochemistry (IHC) were excluded from this analysis. All patients were followed for survival. The protocol was approved by the National Cancer Institute Institutional Review Board.

### Tumor mesothelin expression

Mesothelin IHC was performed on tumor samples obtained using monoclonal antibody 5B2 (Novocastra/Leica, Bannockburn, IL) at 1:40 dilution. One slide was tested for each case. Heat-induced epitope retrieval (20 mins in citrate buffer, pH 6.0) we performed prior to incubation with primary antibody. The detection was performed with Ventana Ultra View detection kit with DAB chromogen. All immunostaining was done using positive and negative controls and results were highly concordant.

Immunohistochemical staining was evaluated by a pathologist (MM) with special expertise in evaluating anti-mesothelin IHC who was blinded to the clinical and molecular data. The positivity (strength of labeling) was assessed as negative (no labeling), weak 1+, moderate 2+, and strong 3+, and the percentage of positive cells was also estimated. High mesothelin expression was defined as mesothelin expression in >25% cells.

### Fluorescence *in situ* hybridization

FISH assays were performed on 5 μm FFPE tumor sections using laboratory standardized protocol with slight modifications. [33] Assessment of *ALK* gene rearrangement was done using LSI *ALK* Dual Color, Break Apart Rearrangement Probe from Visis (Abbott Cat. # 05J89–001). For detection of the *HER2* amplification a FISH probe that consists of two BAC clones (RP11–94L15 and CTD-2248E4) was used. Methodological details of FISH are provided in Supplementary Methods.

### Pyrosequencing

DNA was extracted from FFPE tissue sections using the Qiagen QIAamp DNA FFPE Tissue Kit, according to the instructions of the manufacturer. Pyrosequencing was performed to interrogate the following mutations: *EGFR* exon 19 deletions, point mutations (codons 858, 861, and 863) in exon 21, insertions and point mutations in exon 20 (codon 790) (Ex20), and mutations at codon 719 in exon 18; *KRAS* codons 12, 13, 61; *BRAF* codons 599–601; *NRAS* codons 12, 13, 18, 61; *AKT* codon E17; *PIK3CA* codons 542–546 of exon 9, codons 1043–1047 of exon 20; *HER2* exon 20. Methodological details of pyrosequencing are provided in Supplementary Methods.

### Statistical analysis

The association of dichotomous parameters with mesothelin positivity was determined using Fisher's exact test. The association of race with mesothelin positivity was determined by Mehta's modification to Fisher's exact test. [34] The difference in age between the two mesothelin groups was determined by a Wilcoxon rank sum test. Overall survival time was defined as the time from date of diagnosis of metastatic cancer to date of death or last follow-up. The association between mesothelin and survival was presented using a Kaplan-Meier curve and a log-rank test. The association of mesothelin and survival adjusting for demographic, genetic, and clinical parameters was determined by a Cox proportional hazards model. The association between *KRAS* mutation and mesothelin expression after adjusting for other clinical parameters was determined by logistic regression analysis. All *p*-values are two-tailed and presented without adjustment for multiple comparisons.

## SUPPLEMENTARY METHODS


