# Supplementary material for: High mesothelin expression in advanced lung adenocarcinoma is associated with KRAS mutations and a poor prognosis
Source: Oncotarget. 2015 Apr 13;6(13):11694–703. doi: 10.18632/oncotarget.3429 (PMC4484487; doi:10.18632/oncotarget.3429)
Supplement: Supplementary file 1 [file oncotarget-06-11694-s001.pdf]

SUPPLEMENTARY METHODS

Fluorescence *in situ* hybridization

***ALK* translocation**: For each specimen, minimum 100 qualifying interphase nuclei were scored under BioView Duet-3 fluorescent scanning station. Observed signal pattern for individual cell was scored as positive for rearrangement if 1) green and red signals were split apart by a distance of ≥ 2 signal diameters, or 2) the red signal was observed while green signal was lost (the *ALK* 5′ deletion). Cells were classified as negative if only fused signal(s) representing wild-type *ALK*, or a separate green signal(s) alone were detected. The result of the test with > 10% of positive cells was considered positive.

***HER2* amplification**: The FISH probe used to detect *HER2* gene amplification spans the entire *HER2* gene covering about 300kb (labeled with Sectrum Orange). The alpha satellite centromeric probe for Chromosome 17 (labeled with Spectrum Green) CEP 17 from Abbott Molecular was used as a control for Chromosome 17 ploidy analysis and to calculate ratio of *HER2* gene relative to Chromosome 17. For each specimen, minimum 60 qualifying interphase nuclei were scored (30 by each of two examiners) with a fluorescent microscope equipped with filters to view Spectrum Orange, Spectrum Green, and DAPI. Interpretation of signals was done according to CAP recommendation for breast cancer. [1] Specimen was called positive for *HER2* amplification if the ratio was ≥ 2.2. The result was considered negative for amplification if the ratio of HER-2 signals / CEP 17 signals in the same nuclei was < 1.8. For the ratio ≥ 1.8 and < 2.2, specimen was called equivocal for amplification, and additional 30 nuclei (by each examiner) were scored. If the ratio reproduced was still in the same range, then *HER2* gene amplification result was reported as equivocal.

Pyrosequencing

For identification of point mutations, co-amplification at lower denaturation temperature-PCR (COLD PCR) reactions performed either individually or in a single 96 well microtiter plate (complete gene panel), and the products subjected to pyrosequencing, in an ABI 9700 thermocycler. Following PCR, the products were subjected to pyrosequencing on a Qiagen PyroMark Q24 system. For identification of deletion/insertions, independent PCR reactions were performed with fluorescein labeled primers in the ABI 9700 and the products were analyzed by capillary electrophoresis on an ABI 3130xl Genetic Analyzer.

For *EGFR* analysis, five PCR reactions were designed to interrogate the most commonly occurring mutations including deletion mutations in exon 19 (Ex19del), point mutations (codons 858, 861, and 863) in exon 21 (Ex21), insertions (Ex20ins) and point mutations in exon 20 (codon 790) (Ex20), and mutations at codon 719 in exon 18 (Ex18). Following PCR, the products of the Ex20, Ex21 and Ex18 reactions were subjected to pyrosequencing on a Qiagen PyroMark Q24 system. [2] The products of Ex19del and Ex20ins were analyzed for the presence of microdeletions and microinsertions, respectively, by capillary electrophoresis. The capillary electrophoresis methods were modified from Pan Q. et al [3] and Su Z. et al. [4] For *BRAF* mutation analysis, a single primer set targeting V600, and encompassing codons 599–601 of the *BRAF* gene, was employed, and the PCR product was analyzed by pyrosequencing. [5]

For *KRAS* mutation analysis, two sets of primers from the Qiagen "PyroMark™ *KRAS* v2.0 test", one encompassing codons 12 and 13, and the second encompassing codon 61, were employed, and the PCR products were analyzed by pyrosequencing. For *NRAS* mutation detection, two sets of primers encompassing codons 12, 13, and 18 of the *NRAS* gene, and the second encompassing codon 61, were employed (design available on request), and the PCR products were analyzed by pyrosequencing. For *AKT* mutation detection, a single primer set targeting codon E17 was employed, and the PCR product was analyzed by pyrosequencing. [6] For *PIK3CA* mutation detection, two sets of primers, one encompassing codons 542–546 of exon 9, and the second encompassing codons 1043–1047 of exon 20, were employed, and the PCR products were analyzed by pyrosequencing. For *HER2* mutation analysis, a single primer set encompassing the most common region of activating insertions in exon 20 was employed, and the presence of insertions was assessed by capillary electrophoresis.

REFERENCES

1. Wolff AC, Hammond ME, Hicks DG, Dowsett M, McShane LM, Allison KH, Allred DC, Bartlett JM, Bilous M, Fitzgibbons P, Hanna W, Jenkins RB, Mangu PB, et al. Recommendations for human epidermal growth factor receptor 2 testing in breast cancer: American Society of Clinical Oncology/College of American Pathologists clinical practice guideline update. J Clin Oncol. 2013; 31:3997–4013.
2. Chowdhuri SR, Xi L, Pham TH, Hanson J, Rodriguez-Canales J, Berman A, Rajan A, Giaccone G, Emmert-Buck M, Raffeld M, Filie AC. EGFR and KRAS mutation analysis in cytologic samples of lung adenocarcinoma enabled by laser capture microdissection. Mod Pathol. 2010; 25:548–55.
3. Pan Q, Pao W, Ladanyi M. Rapid polymerase chain reaction-based detection of epidermal growth factor receptor

gene mutations in lung adenocarcinomas. J Mol Diagn. 2005; 7:396–403.

4. Su Z, Dias-Santagata D, Duke M, Hutchinson K, Lin YL, Borger DR, Chung CH, Massion PP, Vnencak-Jones CL, Iafrate AJ, Pao W. A platform for rapid detection of multiple oncogenic mutations with relevance to targeted therapy in non-small-cell lung cancer. J Mol Diagn. 2011; 13:74–84.
5. Xi L, Arons E, Navarro W, Calvo KR, Stetler-Stevenson M, Raffeld M, Kreitman RJ. Both variant and IGHV4–34-expressing hairy cell leukemia lack the BRAF V600E mutation. Blood. 2012; 119:3330–2.
6. Mohamedali A, Lea NC, Feakins RM, Raj K, Mufti GJ, Kocher HM. AKT1 (E17K) mutation in pancreatic cancer. Technol Cancer Res Treat. 2008; 7:407–8.
